# Fine-scale topographic influence on the spatial distribution of tree species diameter in old-growth beech (*Fagus orientalis* Lipsky.) forests, northern Iran

**DOI:** 10.1038/s41598-022-10606-0

**Published:** 2022-05-10

**Authors:** Maryam Fazlollahi Mohammadi, Brian Tobin, Seyed GholamAli Jalali, Yahya Kooch, Rachel Riemann

**Affiliations:** 1 UCD Forestry, School of Agriculture and Food Science, University College Dublin, Dublin, Ireland; 2 Faculty of Natural Resources & Marine Sciences, Tarbiat Modares University, 46417-76489 Noor, Mazandaran Iran; 3 UCD Earth Institute, University College Dublin, Dublin, Ireland; 4 USDA Forest Service, Northern Research Station, c/o USGS, 425 Jordan Road, Troy, NY 12180 USA

**Keywords:** Ecology, Ecosystem ecology, Environmental impact

## Abstract

The Hyrcanian forest in northern Iran is threatened by human use and encroachment and has suffered degradation in some areas. The forest has been declared a World Heritage Site and management in the region is shifting from timber production to conservation. There is considerable interest in developing a greater understanding of these diverse forest communities to inform forest management and multiple use plans to maintain the diversity and resilience of these forests. The Hyrcanian forest is characterized by a complex topography of catenas ranging up mountain slopes. Topographic gradients greatly influence microhabitat conditions which in turn impact tree distribution. To date there has been limited research on the impacts of this diverse topography on the spatial distribution of tree species and tree diameters in Hyrcanian forests. Such information is necessary to better understand the regional traits of tree diameters in these natural mixed temperate forests before forest management occurs. We examined the influence of the area’s catena topography on the spatial pattern of tree species and on species stand structure in terms of tree diameter distribution. To quantify these dynamics, we conducted a complete enumeration inventory of all trees with dbh >12 cm within a 7.947 ha study area that included three C-shaped (concave) and three V-shaped (convex) catenas. Geostatistical variogram analysis and Clark and Evans aggregation index were utilized to study the spatial distribution of tree diameters. Beech, alder, hornbeam, linden and Persian maple exhibited clustered patterns, and sour cherry, ash, and oak exhibited random patterns. Geostatistical analysis clearly revealed the substantial influence of catena topography on the diameter distributions of alder and linden, more subtle influence on the diameter distributions of beech, and a possible influence on Persian maple, providing valuable insight into stand structure over neighborhood-based indices alone. Alder and linden both exhibited strong spatial structure in their diameter distributions (56% and 86%, respectively) where their diameter was strongly correlated with trees within 108 m and 83 m, respectively, sharing more similar diameters to each other than trees beyond that distance. Beech, maple, and hornbeam exhibited very weak if any spatial structure over short distances. These findings can be used to support the alignment of forest management practices in managed Hyrcanian forests with goals of protecting and maintaining biodiversity and sustainable forest ecosystems, and to inform geospatial modeling of species diameter distributions in areas where a complete stem-map is not feasible.

## Introduction

Environmental factors impact both individual species distribution patterns across a landscape as well as the structure of plant communities^1^. In mountain ecosystems, topographic features including aspect, slope angle, and elevation above sea can affect site parameters like sunlight distribution, humidity, and nutrient availability, each of which can be primary determinants of local species distributions, as well as plant growth and mortality patterns^2^. It is thus common for sites with different topographic characteristics to contain different forest types^3^, and each forest type may have its own separate type of understory, floral diversity, and spatial distribution of associated plant communities^4^. Analysis of the spatial variation and structure within forests can provide valuable information for understanding ecosystem-level processes such as the relative success or sustainability of regeneration and to detect early signs of stress or a lowering of resilience to disturbances such as climate change.

The Hyrcanian forests of northern Iran stretch from Astara in the northwest along the southern coast of the Caspian Sea as far as Gorgan in the northeast, and from 20 m above sea level to approximately 2,800 m up the Alborz mountains. Classified as Hyrcanian mixed forests, they are composed of a mixture of uneven-aged deciduous broadleaf tree species^5^. These Hyrcanian forests are among the Earth’s oldest and contain a unique assemblage of endemic tree species. They are also under significant threat from growing urbanization and utilization pressures and since 2019 have been identified by UNESCO as a world heritage site. These forests are considered to be relics of ancient deciduous broadleaf temperate forest, stretching from SE central Europe to SW Asia since the Arcto-Tertiary era, that retreated during the Quaternary glaciations and then expanded again post-glaciation^6^. These forests have experienced minimal anthropogenic disturbance and typically include trees ranging to their maximum ecological age, large amounts of standing dead and coarse woody debris, and a stand structure that is both horizontally and vertically heterogeneous. Although covering only a small portion of the country now (1.1%), 44% of the vascular plants known in Iran are found in the Hyrcanian region. The catena topography of this region influences both regional and micro-climate conditions which can have a large effect on tree species distribution and environmental processes^5^. The management objective of Hyrcanian forests controlled by the Iranian Forests, Range, Watershed, and Management Organization (FRWO) is to maintain the current forest stand composition, protect its level of biodiversity and avoid any shift in the composition of commercial forest types^7^ (https://whc.unesco.org/en/list/1584).

The spatial distribution of tree populations in a particular forest ecosystem is typically characterized in terms of patterns in tree location, tree size, and the mingling of different tree species^8^. Tree population structure for a single species can be considered in terms of the spatial distribution of tree stems, diameters, and age^9^. When accurately characterized, distribution patterns of forest structure directly reveal how trees are aggregated or dispersed in the landscape. Patchiness, which shows how much trees are clumped or scattered, is closely related to how that species uses resources, the success (or otherwise) of its reproductive strategy (dispersal habits and ability to utilize gaps of different sizes), levels of competition present, and how the species has been used/exploited as a resource^10^. How mature a tree population is, its survival viability, competition ability within the community, and the nature of its ecological niche all affect tree diameter distribution, either directly or indirectly^11^. Li et al.^9^ opine that tree species in a mixed stand develop interspecific and intraspecific differences in size, species mingling, and distribution patterns, and that these are the three most important characteristics of population structure to assess for better understanding an ecological niche, its degree of maturity, and the survival viability of its tree communities. Such knowledge would better inform management towards supporting resilience of mixed-species, multi-structured stands.

In a previous study, we investigated species composition and diversity (including trees, regeneration patterns, and soil traits and profiles) in relation to landform and slope position. The results of these studies revealed that the number of trees for almost all species increased from the mid-slope (back slope position) to downslope positions (such as foot slope and toe slopes in small-scale topographic gradients)^12,13^, corresponding to the nutrient and moisture availability at these positions^14,15^. In catena landforms, some species like hornbeam were separated from other trees and tended to create pure groups. We hypothesized that if topography and landform can affect the tree composition, diversity, and soil traits, it may also influence tree spatial distribution and forest structure.

Topographic gradients influence the composition and function of forest communities, caused by diversity in microsite and availability of resources that are associated with changes in topography. Harms et al.^16^ and Gunatilleke et al.^17^ conducted such studies in neo-tropical forests. Gunatilleke et al.^17^ found that 79% of the species were associated with topographically defined habitats in the Sinharaja forest (Sri Lanka). In temperate regions, topography has been observed to influence forest stand composition and coarse woody debris occurrence in North America^18^, Europe^3^, and also in Iran^12,15,19–21^. In the Hyrcanian forests, topography has been observed to affect most soil properties^14^, which subsequently affect vegetation composition and diversity^12,15^. In these forests, the northern slopes of the Alborz Mountains have the most productive sites. On these north-facing slopes beech, a shade-tolerant species, is the dominant tree. This is in contrast to the oak-dominated forests which are common across the south-facing slopes^5^.

**Figure 1:**
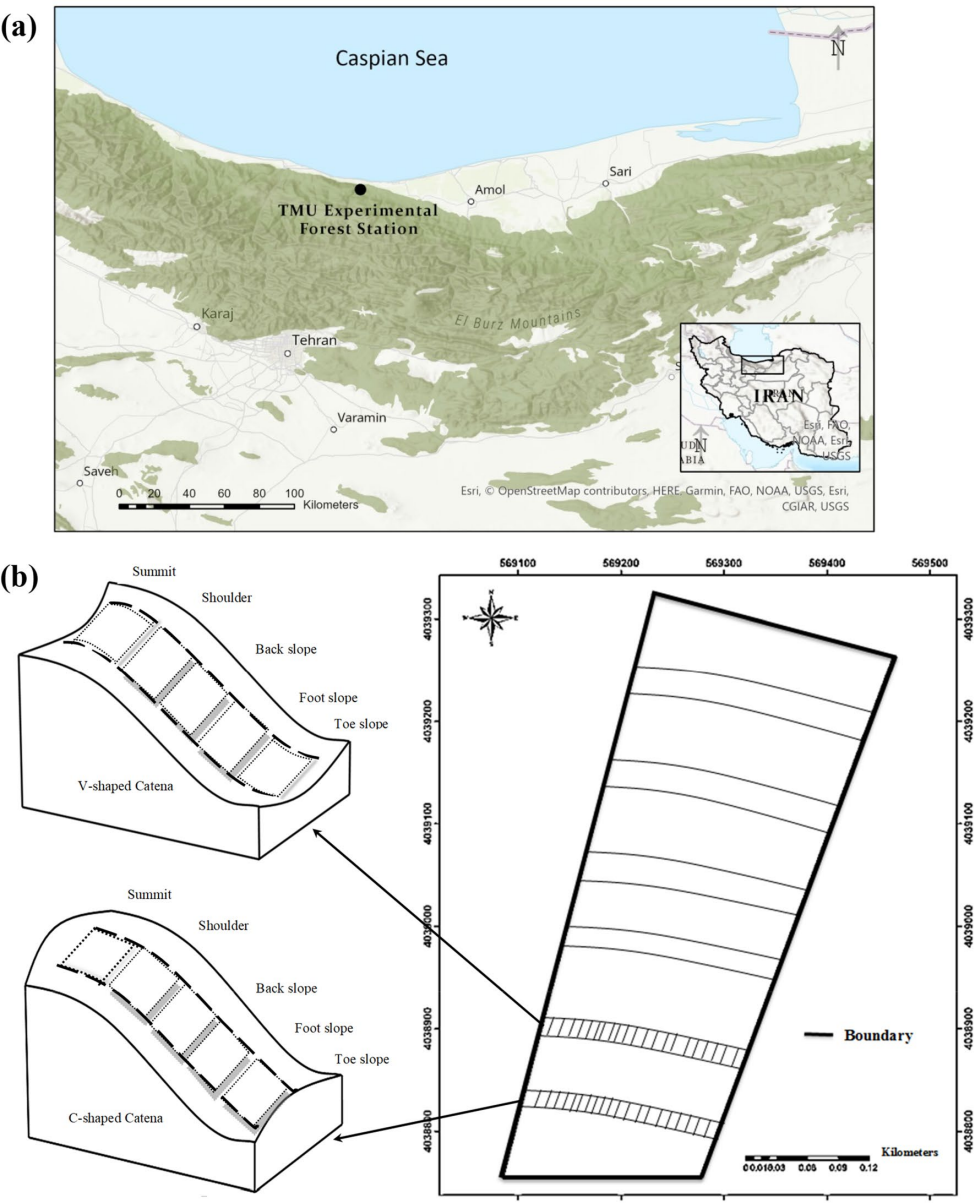
Location of the study area (**a**), and a diagram illustrating orientation, and the shape of the two major catena types, as well as their approximate locations in the study area (**b**). Figure (**a**) was produced using ArcGIS Pro version 2.8.6, https://pro.arcgis.com/en/pro-app/2.8/get-started/get-started.htm, and Figure (**b**) was drawn using Microsoft Word 2013 V 15.0.5085.1000, https://www.microsoft.com/en-ie/microsoft-365/previous-versions/microsoft-word-2013, and was first published in Fazlollahi Mohammadi et al. (2017).

The Northern Hyrcanian forests occur in a topographically dissected landscape. The longer and larger SW to NE sloping landscape is crossed by convex and concave catenas that slope in approximately an E-W direction (see Fig. 1). Little is known regarding how the spatial patterns of trees and their diameter distributions in the Hyrcanian forest are affected by these catena landforms. Previous studies of these forests have investigated the effect of landform on understory plant communities and tree species composition, biodiversity, and regeneration^12,15^. The aim of this study was to examine the spatial distribution of tree diameters in these topographically dissected landscapes, first to create a baseline dataset with which later developments may be compared (as an indicator of change), and second to better understand forest functioning and stand dynamics (with a view to informing conservation management). In this study, we apply both an aggregation index^22^ and geostatistical variogram analysis to assess tree species and diameter distribution patterns and examine their relationship to the catena topography of the natural Hyrcanian mountain forests in northern Iran. The relatively low level of anthropogenic disturbance in these Hyrcanian forests provided an opportunity to examine the characteristics of a mature, temperate, mixed species forest system that is currently in a self-sustaining state^23^. Tree size distribution is a common analog for examining age structure and has been used to understand population viability^9^. As Pelissaria et al.^24^ point out, a deficiency of ecological information often leads to the application of new techniques for analyzing spatial variations in forests. The better we understand the environmental factors influencing diameter distribution the better our understanding and management of woody species in these mixed Hyrcanian forests will be. In addition, it remains unknown how species identity and stand structural composition drive effects like overyielding, crown extension and mortality, so investigations into interactions between compositional and structural diversity in mixed, uneven-aged forests is highly relevant for the development of silvicultural approaches to mixed-species management^25^. Studying the distribution of tree species within this topographically complex landscape will facilitate more accurate ecosystem evaluation, e.g. as new pathogens potentially cause selective mortality of trees or new management scenarios are considered for increasing resistance and resilience against natural and anthropogenic disturbances^7^.

## Material and methods

### Study area

We collected data from the Tarbiat Modares University (TMU) Experimental Forest Station, located in the Hyrcanian forest of the Mazandaran province in northern Iran, between 36°31′56″N and 36°32′11″N latitude and 51°47′49″E and 51°47′56″E longitude (Fig. 1a). This forest is considered to be relatively unthreatened by anthropogenic activities and to be representative of the original Hyrcanian plain forest situated south of the Caspian Sea^26^. The regional climate is humid-temperate (based on the Köppen classification), with mean annual temperature of 10.5 °C, rainfall of 1030 mm, and relative humidity of 75.2%. The parent material in the study area consists of limestone and dolomite limestone^5^, which originates from the upper Jurassic and lower Cretaceous periods. The soil classification is Typic Endoaqualfs in this region^27^. The average cation exchange capacity in the top soil ranges from 10 to 12 cmol( +)kg^−1^, pH ranges from 6.0–7.5, and soil textures range from silty clay loam to loam^13^. The depth of the solum varied from 87 to 150 cm depending on slope position. All soil profiles within the study area consisted of O, A and B (Bt or Bht) horizons, with the soil profiles at the downslope positions classified as more developed^13^. The forest is described as a multistoried, multi-aged beech stand, dominated by *Fagus orientalis* Lipsky (oriental beech), *Carpinus betulus* L. (hornbeam), *Alnus subcordata* C.A.Mey. (alder), and to a lesser extent, *Acer velutinum* Boiss. (Persian maple), and *Tilia platyphyllos* Scop. (linden). Other tree species in the study area include *Quercus castaneifolia* C.A.Mey. (oak), *Cerasus avium* Moench. (sour cherry), *Fraxinus excelsior* L. (ash), and *Acer cappadocicum* Gled. (Cappadocian maple). Oriental beech is generally found from 800 to 1800 m, becoming more dominant at higher elevations upslope^12^.

### Study site and topographic data

**Figure 2:**
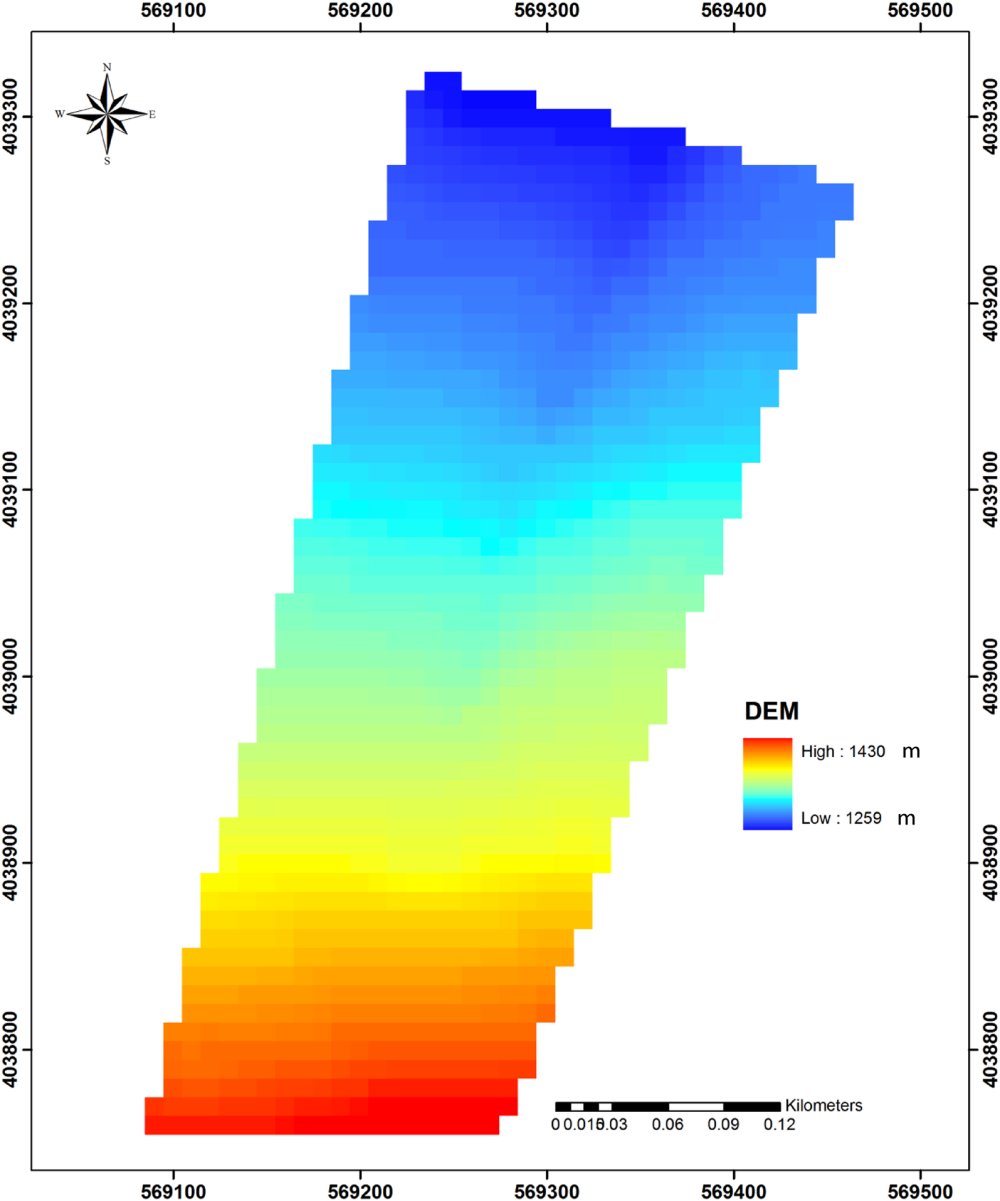
Digital Elevation Model (DEM) map of study area.

**Figure 3:**
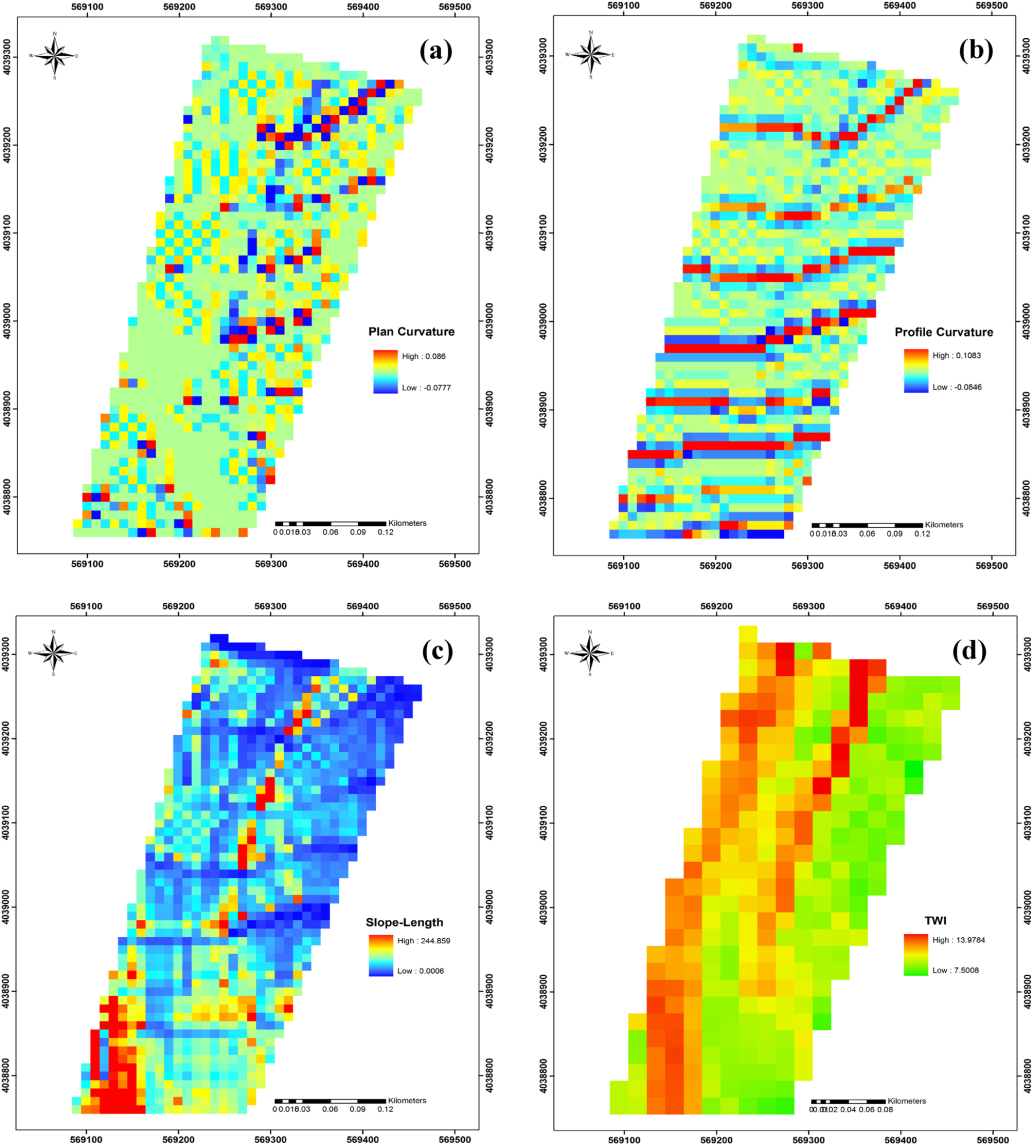
Plan curvature map of the study area (**a**), the profile curvature map of the study area (**b**), the slope length of the study area (**c**), topographic wetness index for the study area (**d**). The locations of all six catenas are clearly visible in the profile curvature map, somewhat visible in the slope length map, and partially visible in the plan curvature map. This calculation of the topographic wetness index appears not to be affected by the catena features.

In the summer of 2013, we established a 7.947 ha study site that encompassed six catenas with two distinct shapes—including three catenas with a convex slope (called V-shaped hereafter) and three catenas with a concave slope (called C-shaped hereafter) (Fig. 1a and b). The location of the study site was chosen because its catenas were all similar in aspect (northeast), slope (about 23%), and length (about 150 m) and thus reduced the extent of topographical variation. Catena characteristics were determined using digital elevation model (DEM) data with a resolution of 10 mm^28^ (Fig. 2), from which was calculated the primary properties of plan curvature (Fig. 3a), profile curvature (Fig. 3b), and slope length (Fig. 3c). Catenas are regularly occurring micro-topographic features in these landscapes that are not visible in the 10-m DEM data, but are clearly visible in the profile curvature map. In addition, the secondary property of Topography Wetness Index (TWI) (Fig. 3d) was also calculated^29^ in SAGA-GIS^30^ (System for Geoscientific Analyses) software.

### Tree data

We conducted a complete enumeration inventory to determine the spatial distribution of trees in the TMU Experimental Forest Station (covering an entire area of 7.947 ha, see Supplementary Figures S1 and S2). The diameters of all trees with a diameter at breast height (dbh) ≥ 12 cm were measured (1.3 m from the ground) using calipers. Following the procedure outlined by Moeur^31^, a reference point was chosen in the first catena (as the start point), and its coordinates were recorded using a high sensitivity hand-held GPS (Garmin 60cx)^31^. Then, distances between the reference point and all trees clearly visible from the reference point were recorded using a laser device (Bosch Laser Distance/Range Measurer). We also recorded the azimuth bearing of each tree from the reference point using a digital compass^32^. Then, another reference point was chosen, and the same surveying procedure was followed until all trees within the area were marked. In total, 50 reference points were located for stem mapping. The coordinates of each tree were calculated using the coordinates of the reference points, and the distances and azimuth values from the reference points^33^. A total of 1,498 stems were measured, belonging to 9 species. Catena locations were identified in the field using the GPS and then compared with DEM-derived elevation and slope measurements to verify field observations. Comparisons were made at each of the five slope positions (Fig. 1b). Tree location (x, y), species, and dbh were used in this study.

Summary statistics including mean dbh (cm), and tree density (stems ha^−1^) were calculated for the inventory dataset. We also used Kolmogorov–Smirnov tests for assessing the normality of tree stocking distribution in diameter classes. Spatial patterns in tree locations and tree diameters were examined using Clark and Evans^22^ aggregation index and variogram analysis. Each of the nine species was examined separately – beech, alder, hornbeam, linden, Persian maple, Cappadocian maple, oak, ash and sour cherry.

### Clark and Evans aggregation index

Clark and Evans^22^ developed an aggregation index that calculates a single value *R* to describe the variability of tree locations. In this study we used the equation presented in Pommerening^34^ (Eq. 1):

1 $$R=\frac{{\bar{r}}_{observed}}{E\left(r\right)}\quad {\mathrm{where}}\quad E\left(r\right)=\frac{1}{2*\sqrt{\frac{N}{A}}};$$

$$R\in \left[0, 2.1491\right]$$

In this equation, the average distance measured from each tree to its nearest neighbor (${\bar{r}}_{observed}$) is compared to the average nearest-neighbor distance in a stand with completely random tree locations ($E\left(r\right)$), which is calculated as a function of forest area (*A*) and number of trees present (*N*). If $R>1$ tree distribution tends toward regularity; if $R=1$ tree distribution is completely random (Poisson process); and if $R<1$ tree distribution is in a clumped pattern^34^. The aggregation index was calculated using SpPack version “st0292” software.

### Geostatistical variogram analysis of dbh spatial variability

A variogram is a graphical plotting of the similarity of a variable (here tree diameter) measured at points in the landscape, as a function of the distance (in meters) between pairs of those data points. The variogram is a central tool in geostatistics^35^. Each plotted value in the variogram, γ($h$), is equal to half of the average squared difference between each data pair within a specified lag distance $\left(h\right)$ from each other, (Eq. 2)^36^.

2 $$\gamma \left(h\right)=\frac{1}{2N\left(h\right)}\sum_{i=1}^{N\left(h\right)}{\left[\left(z\left({x}_{i}\right)-z\left({x}_{i}+h\right)\right)\right]}^{2}$$

The variogram provides valuable information about both the range of spatial correlation present, as well as the strength and direction of that correlation. Several important characteristics of the variogram are the sill, the range, and the nugget. The sill defines where the average squared difference in dbh between the pairs is greatest—i.e. the maximum variogram value γ($h$). The range defines that distance (meters) where the variogram reaches the sill—i.e. that distance over which tree dbh values are more similar than their maximum variation. Beyond the range distance, dbh values are no longer spatially correlated^37^. The nugget defines the y-intercept (always positive) of the variogram. The closest calculated variogram value is from the smallest distance bin. The nugget value is typically identified through extrapolation of a model chosen to describe the shape of the variogram. A nugget larger than zero is caused by measurement errors and sources of variation at distances smaller than the lag distance used^38^. Even if the variation for a particular variable is in reality locally spatially structured, the calculated variogram could still be pure nugget or large nugget if the sampling scale isn’t sufficiently sensitive to pick up that local spatial structure. One parameter set by the user is the lag distance (*h*), which defines the size of the distance bins used to calculate each $\gamma \left(h\right)$ value in the variogram and is limited by the sampling intensity of the available data. As suggested by Oliver and Webster^39^, preliminary investigation of the data is typically required to identify the most effective lag distance, and whether a directional component to the observed spatial structure is present, a situation in which observed spatial structure is different in various directions. In this study, variograms were calculated using GS + Ver. 10 software.

## Results

**Figure 4:**
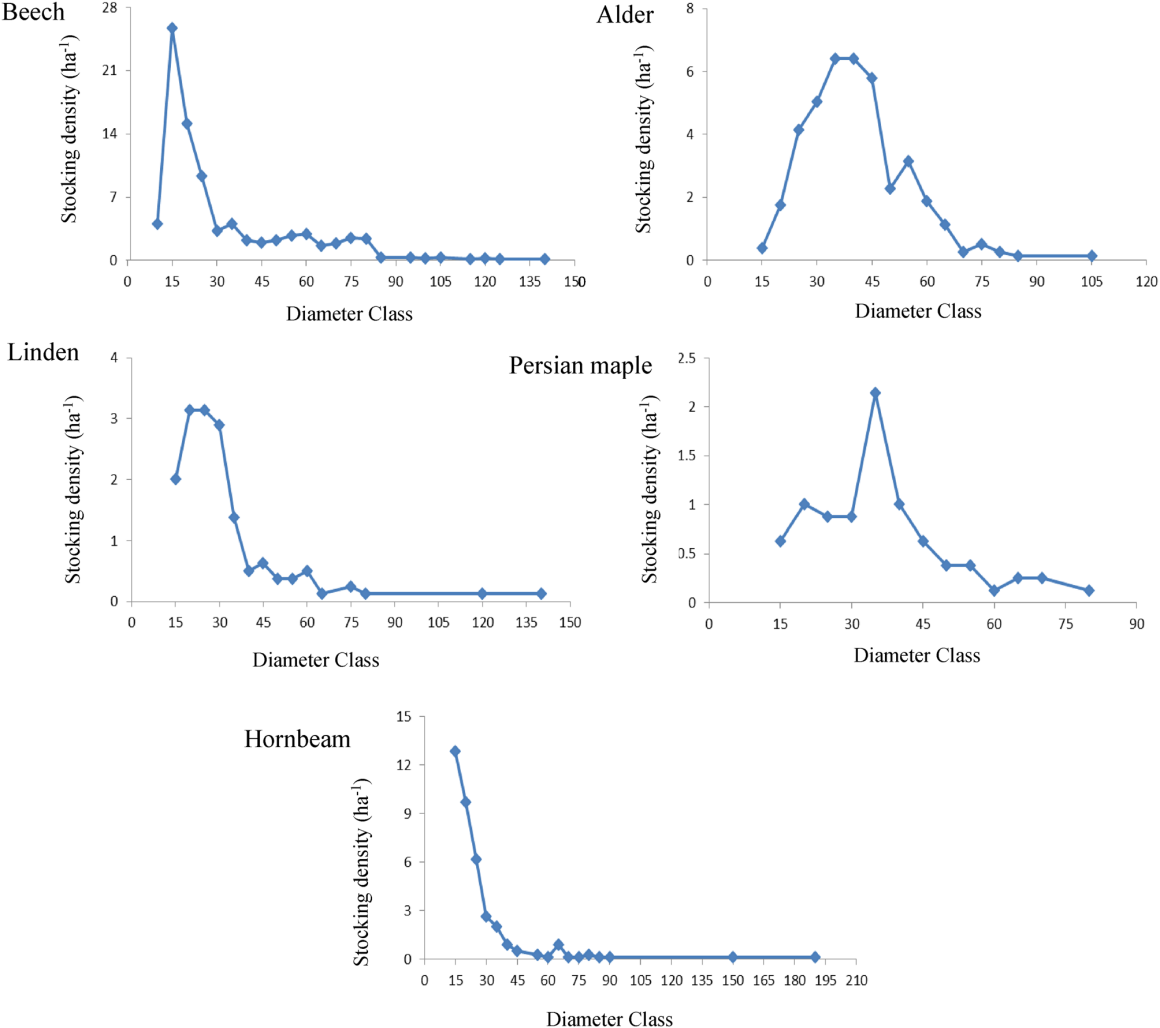
Stocking density of trees by diameter class, for beech (*Fagus orientalis*), alder (*Alnus subcordata*), linden (*Tilia platyphyllos*), Persian maple (*Acer velutinum*) and hornbeam (*Carpinus betulus*), using dbh-classes of 5-cm bins.

**Figure 5:**
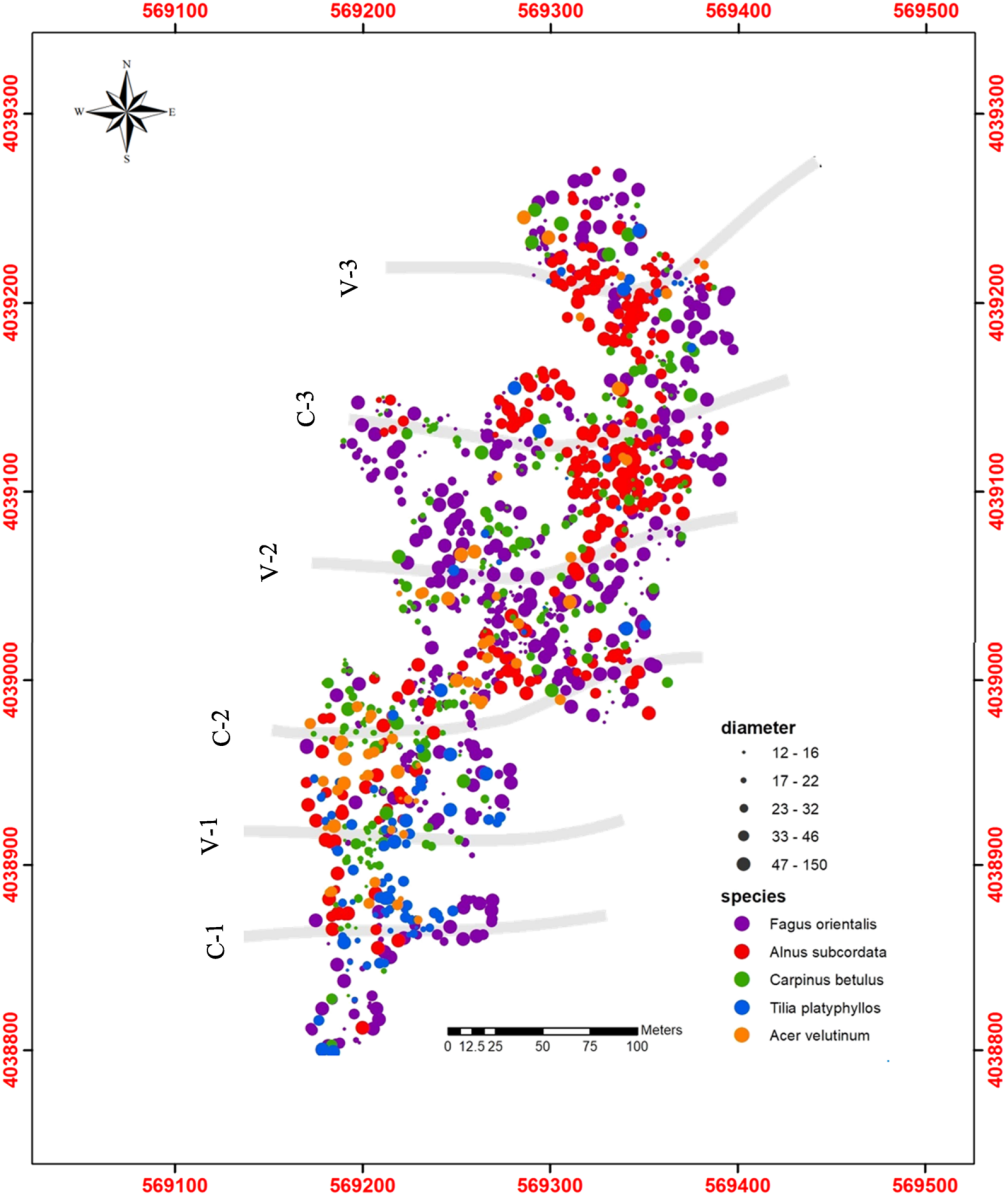
Spatial distribution of five species by diameter class; beech (*Fagus orientalis*), alder (*Alnus subcordata*), linden (*Tilia platyphyllos*), Persian maple (*Acer velutinum*), hornbeam (*Carpinus betulus*). The isolines are catenas (six C- and V-shaped) derived from the profile curvature map.

We encountered a total of 9 tree species in the study area. The overall stocking density in this forest is relatively low at 189 stems ha^−1^. The number of trees per species ranged from 3 to 669 stems in the 8-ha study area. Beech (669) had the highest density, followed by alder, hornbeam, linden, Persian maple, Cappadocian maple, oak, ash, and cherry (Table 1). Beech, hornbeam and linden exhibited reverse J-shaped diameter frequency plots (Fig. 4), indicating dominance of lower diameter trees in the study area, a characteristic of forests that have scant history of harvesting or management. Diameter distributions for alder and Persian maple demonstrated more bell-shape curves (were normally distributed) with righthand skewness. For four of the species (Cappadocian maple, oak, ash, and cherry), there were not enough trees present to draw diameter distribution graphs (less than 20 trees). Diameter distribution maps of the study area for the five most dominant species are presented in Fig. 5, along with the location of the six catenas as derived from the profile curvature map. The statistics of dbh by species including maximum, mean, minimum, skewness, standard deviation, kurtosis and coefficient of variation are presented in Table 2. Alder exhibited the largest mean diameter (40.49 cm) and hornbeam (24.71 cm) the smallest. Beech, hornbeam, and linden distributions were all skewed toward lower diameter classes, while Persian maple and alder exhibited distributions with much less skewness (Table 2, Fig. 4).

**Table 1:** Spatial distribution characteristics in the studied area.

Species name	Common name	Number of trees	Density (trees ha^−1^)	Mean distance to nearest neighbor (m)	Clark and Evans index
*Fagus orientalis*	Beech	669	84.18	3.91	0.47
*Alnus subcordata*	Alder	306	38.5	4.68	0.49
*Carpinus betulus*	Hornbeam	285	35.86	7.25	0.38
*Tilia platyphyllos*	Linden	124	15.6	5.99	0.45
*Acer velutinum*	Persian maple	69	8.68	8.42	0.5
*Acer cappadocicum*	Cappadocian maple	19	2.39	16.15	0.49
*Quercus castaneifolia*	Oak	15	1.88	27.97	1.39
*Prunus avium*	Cherry	8	1.00	24.55	–
*Fraxinus excelsior*	Ash	3	0.37	125.38	–

**Table 2:** Summary statistics describing species’ diameter at breast height (cm).

Species name	Mean	Minimum	Maximum	Standard deviation	Skewness	Kurtosis	Coefficient of variation (%)
*Fagus orientalis*	31.5	12	150	22.9	1.6	2.6	72.6
*Alnus subcordata*	40.4	12	106	13.4	0.9	1.6	33.1
*Carpinus betulus*	24.7	12	190	17.9	4.9	34.1	72.4
*Tilia platyphyllos*	31.5	12	140	19	2.8	11.5	60.1
*Acer velutinum*	36.1	12	82	14.5	0.7	0.7	40.1

Results from the Clark and Evans aggregation index indicated a random distribution for oak (1.39), however, most of the dominant tree species were non-randomly distributed within the study area. Beech (0.47), alder (0.49), linden (0.45), Cappadocian maple (0.49) and Persian maple (0.5) all exhibited clustered distributions, with hornbeam (0.38) reporting the most clustering. Ash and cherry were considered to have too few trees to calculate a reliable aggregation index value. Mean distance to the nearest neighbor of the same tree species is shown in Table 1. The highest nearest neighbor distance was seen for ash (125.38 m) and the lowest for beech (3.91 m).

**Figure 6:**
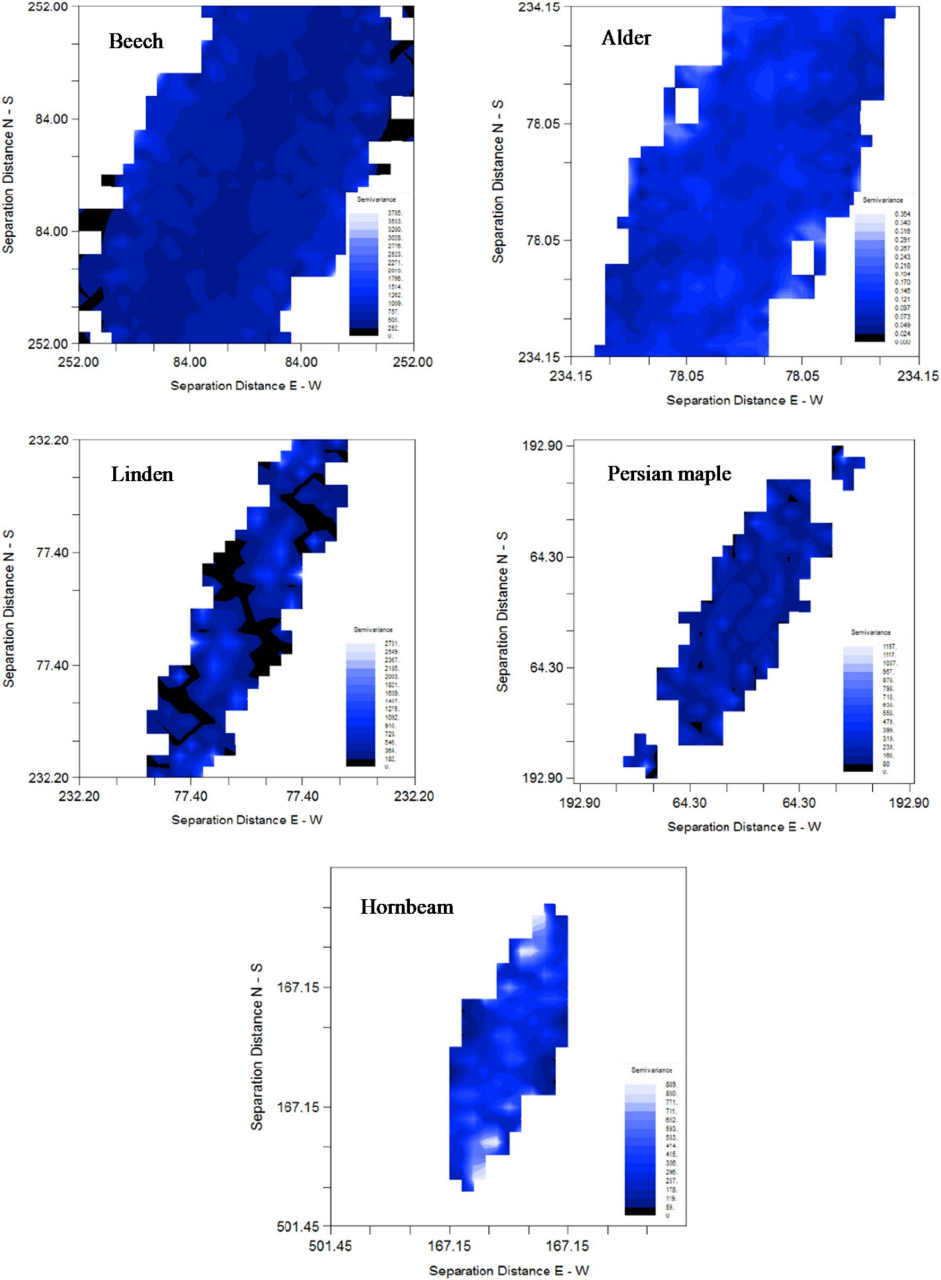
Surface variogram of anisotropy for beech (*Fagus orientalis*), alder (*Alnus subcordata*), linden (*Tilia platyphyllos*), Persian maple (*Acer velutinum*) and hornbeam (*Carpinus betulus*). A surface variogram is used to detect the presence and direction of anisotropy in the data. If anisotropy is present, the variogram calculated for that species will be different when measured in different directions.

Multi-directional or isotropic variograms for these five most common species are presented in Fig. 6, and their characteristics are summarized in Table 3. Alder and linden both exhibited strong spatial structure in their diameter distributions (56% and 86%, respectively), with trees closer than 108 m and 83 m, respectively, more similar to each other than trees beyond that distance. Beech, maple, and hornbeam exhibited very weak if any spatial structure over short distances. Notable in four of the variograms (for all apart from hornbeam) is that the sill never settled down to a single semi-variance value but exhibited a cyclic pattern around the sill. This characteristic was most clear in the variograms of alder and linden. This cyclic pattern is consistent with the periodic presence of catenas across the study area C. The study area encompasses a complex topography, with both an overall upslope-downslope trend in the SW-NE direction and ridge-valley structures consisting of multiple catenas crossing in an E-W direction across that general slope. When spatial structure thus differs in two separate directions, the impacts arising from one of the structural systems may be masked in an isotropic variogram. Surface variograms for the five species provided an initial look at the possibility of directional differences in spatial structure (Fig. 6). Because of the shape of the studied area, the surface variograms include greater distances in the study area’s long direction (NE-SW), than in the short direction (SE-NW). The surface variograms for Persian maple or hornbeam indicated no clear directional pattern, and it is likely that the apparent pattern in the surface variogram for linden (black areas in Fig. 6) is due to insufficient data in those directions. The surface variogram for beech exhibited greater spatial structure (lower variance) in the upslope-downslope (NE-SW) direction than along-the-catena (E-W). This suggests that calculating directional variograms for beech could reveal different spatial structures that are otherwise muffling each other in the isotropic variogram. For example, a directional variogram in the NE-SW direction (which would be parallel to the general DEM elevation and across the catenas), could reveal a stronger cyclic structure (higher amplitude) than was exhibited in the isotropic variogram for beech. Directional variograms require more data because they are calculating the variance between neighbors over a smaller direction window. The surface variogram for alder exhibited a more subtle anisotropy in the direction of the catenas.

**Table 3:**
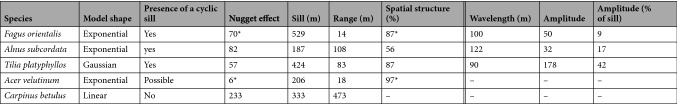
Variogram characteristics for five tree species.

Values typically assessed from variogram analysis for interpretation with respect to the landscape are the nugget, sill, range and spatial structure. However, the wavelength and amplitude of the variogram sill instead of a single average number are important in this study where the unusual cyclical sill patterns indicate a relationship between dbh and the topography. *Reflects values considered over-fitted by the automatically generated model.

## Discussion

The spatial distribution of plants in mountainous areas is controlled by three main topographic factors, elevation, aspect and slope^40^. The topographic heterogeneity in these regions usually causes variation in soil properties within what may otherwise be a relatively uniform climatic region^41^. Topography influences some forest dynamics^42^ and nutrient cycles^43^ by altering site attributes such as drainage regimes and soil properties which are correlated with tree species distribution in forests^30^. Beaty and Taylor^44^ reported that slope, aspect and position within a site resulted in considerable variation in forest structural composition, and Castilho et al.^45^ have even used topography to estimate live biomass in central Amazonia. Ecological processes may control the timing of disturbances across a landscape, but topography often determines their spatial pattern^46^.

The skewed bell-shaped diameter distribution of alder and Persian maple (Fig. 4) is consistent with a period of strong regeneration of these species after a major windthrow disturbance concentrated on the exposed parts^47^ convex areas of the catena structures^48^ followed by a more recent decline in regeneration perhaps as the available light declined with increasing canopy closure. These results suggest that these two species and their regeneration patterns were affected by the catena topography (both C- and V-shaped), through association with major windthrow events that were seen in both catena shapes but more so on convex catenas. The diameter distributions of beech, hornbeam and linden often exhibit a reverse J-shaped distribution form in mixed forests managed for conservation^49,50^, and this distribution was generally observed here as well.

Examination of the Clark and Evans aggregation index indicated that oak was randomly distributed across the study area. In contrast, beech, alder, linden, Cappadocian maple, Persian maple, and hornbeam all exhibited a clustered pattern in the distribution of tree stems. These results parallel results from Kunstler et al.^51^ who studied the spatial distributions of oriental beech on the Causse du Larzac plateau in France and found similar clustered patterns for beech (the species composition was different, but individual species, e.g. beech, were clustered). Other studies have reported random distribution patterns for oak^52^. Although acorns are heavy seeds, their dispersal is driven by animal activity, in particular by jay species (*Garrulus glandarius*) and this accounts for the randomized distribution pattern^53^. Ghalandaraveshi et al.^54,55^, and others reported clustered distributions over small scales for European beech trees (*Fagus sylvatica*) which have heavy seeds with limited seed dispersal^56^. Clustering over short distances is usually associated with limitations for seed dispersal, higher light intensity in gaps, and competitive interactions between trees. In this study, the Clark and Evans index was calculated over the entire study area (7.947 ha), and clustering at this larger scale was observed for all five of the dominant species; i.e. beech, alder, hornbeam, linden and Persian maple all exhibited clumped distributions. Clustering over broader scales usually results from heterogeneity over the study area or large-scale disturbance patterns^54,57^, both of which were found in the study area.

**Figure 7:**
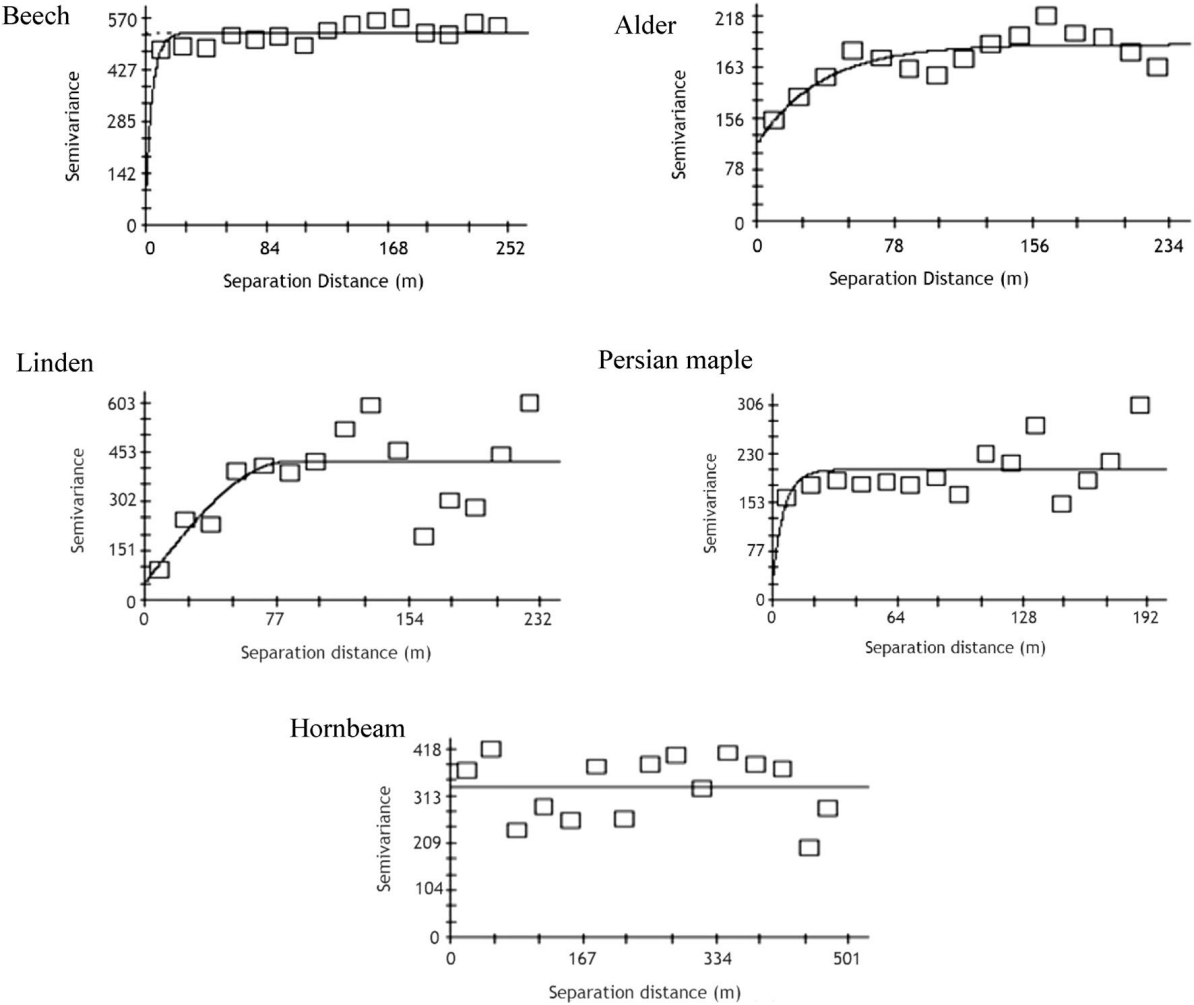
Experimental variogram and the estimated linear model for beech (*Fagus orientalis*), alder (*Alnus subcordata*), linden (*Tilia platyphyllos*), Persian maple (*Acer velutinum*) and hornbeam (*Carpinus betulus*).

Geostatistical analysis results clearly supported earlier observations that tree species in the Hyrcanian forests have distribution patterns related to the catena topography of this region. The spatial structure of diameter distribution differed by species. Some species such as alder, linden, and Persian maple were strongly affected by the catena landform, visible as a cyclic pattern of spatial correlation in the variogram. Beech and hornbeam appeared to be much less affected (Fig. 7). Surface variogram results indicated that the distribution of species diameters is influenced both by general NE-SW elevation gradient and the presence of the E-W catenas (Fig. 6). Natural stands of beech in the Hyrcanian forests have high volume and basal area per hectare^58^, with beech having more total basal area than any other species. In these forests, hornbeam frequently occurs in the beech understory. Although hornbeam was the third most abundant species after beech and alder, it does not represent a large percentage of the forest’s total basal area or standing volume. Beech is a shade-tolerant and mesophytic species and was found with greater frequency on lower-slope positions and on V-shaped catenas. In contrast, species such as hornbeam were observed at a higher frequency on foot slope positions in both catenas^15,20^.

Lan et al.^59,60^ found strong evidence that topographic factors affected tree species distribution patterns in southwestern China, with landform and elevation above sea level being the most valuable of the four topographic characteristics examined. Variation in both convexity (the catenas) and elevation are features of the Hyrcanian forest region topography and also appear to affect both tree species distribution and diameter distribution, suggesting that factors related to specific conditions across the landscapes such as productivity of soil and water availability may be contributing to the observed tree distribution patterns. Both convexity and elevation affect soil moisture, which can affect both the chemical and physical properties of soils, e.g. where V-shaped catenas and lower slope positions contain more moisture and microbial biomass than C-shaped catenas and upslopes^13^. Menendez et al.^61^ observed that soil solution composition depended on soil water content. The calculation of the TWI was apparently unaffected by the catena topographical features (Fig. 3d), and appears to be more heavily influenced by the slope length gradient. However, without ground-truthing against data of fine resolution soil moisture assessments it is difficult to evaluate its accuracy, even if it appears to ignore the topography. This point is made by Kopecký et al.^62^ who demonstrated that the calculation method hugely affects the ecological relevance of calculated TWI values for any given landscape, and in particular depends on how the local slope gradient is specified. Both site heterogeneity and periodic windthrow events associated with the catena topography are likely to be influencing factors in large-scale clustering in this area as the windthrow gaps were concentrated over convex slope areas. It has been reported that Oriental beech forests often develop in three major development stages, each of which can be subject to a wind disturbance regime that results in a mosaic of patches specified by these stages occurring close to each other in space or developing in a cycle over time^63,64^.

Within the catenas, tree species exhibited distributions including an extended range of diameters and generally rapidly declining number of trees within the higher diameter classes for most species. This distribution is typical of old-growth stands^54^. In the case of ash, there were only three individuals in the entire 7.9-ha study area. Ash produces a large amount of annual seed that requires higher light intensity for germination, and its wind-dispersed seeds with broad dispersal patterns typically enable ash to successfully regenerate in large gaps formed by natural disturbances^54,65,66^. In this study, the random distribution of oak, ash, and cherry is probably a consequence of this requirement and the pattern of gaps in this area. The occurrence of relatively few ash trees in the study area, each of which were large remnant trees, could suggest either insufficient quantities of seed to fund regeneration, insufficient gap frequency of adequate size or high browsing pressure from ungulate species^67,68^. Although not wind-dispersed, oak and cherry may be sufficiently common in the Hyrcanian forest to regenerate in available gaps and take advantage of the opportunity for light throughout their life cycle. Lan et al.^60^ pointed out that dispersal by animals is a dominant factor in temperate forests and may also be a factor here. The “continuous and fast-growing” strategy that characterizes ash, oak, and cherry growth patterns allow these species to quickly take advantage of gaps as they present, but requires an element of temporal luck to coincide with a gap when it forms. In contrast, the “stop and go” strategy of beech allows it to take advantage of changing light conditions throughout its lifetime. Szwagrzyk and Szewczyk^69^ observed that seedlings of beech emerge often without light availability, resulting in their random spatial distribution, but that beech seedling survival was strongly dependent on higher light intensities and thus clustering in those areas. Beech responds quickly with increases in available light both with growth and lateral crown expansion that inhibits competitors^70^. These growth habits are consistent with the clumped spatial distribution patterns of beech elsewhere within the Hyrcanian forest region^49,71^.

In the study area, the complexity of the catena topography very likely contributed to both the clustered patterns observed in the aggregation index of the six tree species and the strong local spatial correlation and cyclical sill pattern for alder and linden in the variogram results. In our previous studies of this area, topography also resulted in the variation of tree species, ground vegetation, and soil properties^12,14,15^. The influence of topography (including slope, aspect, and steepness) on forest stand composition has also been observed in both the oak-dominated forests of western Iran^21^ and the beech forests of northern Iran^20^. The study by Valipour et al.^27^ observed a topographic influence on tree diameter as well. High values for nugget effects like the ones observed in this study may be typical in forested ecosystems, and contributed to by less predictable events like fire, windthrow, and disease. Spatial correlation may be present, however, even with a large nugget effect. The variogram for alder (Fig. 7) showed a strong local spatial correlation with a range of spatial correlation of tree diameter up to 108 m. Linden also showed strong local spatial correlation over a range of up to 83 m*.* The range of local spatial correlation for beech and Persian maple diameters was less than 20 m if it was present at all, while hornbeam showed no spatial correlation of tree diameter (Table 3). The variogram analysis of tree diameters also identified a scarcity of very small trees indicative of healthy successional regeneration in the ground and understory. This reflects the low stocking density (on average 189 trees ha^−1^) and was apparent on the ground. Although only trees of dbh > 12 cm were enumerated, as can be seen in the Supplementary Figures S1 and S2, there was little in the way of early-stage regeneration, even in gap areas. This anomaly in the younger age structure is probably indicative of excessive browsing pressure from deer, goats and other ungulate species, as noted by Pourmajidian et al.^68^ and Soofi et al.^72^.

Geostatistical methods, like many statistical methods, assume that the data are error-free. The potential impact of the measurement and sampling errors and outliers on results in this study is unknown. If not incorporated into the statistical method, the potential error must be considered in the interpretation of the results^48^. Measurement error could contribute to a larger nugget variance than might otherwise be true; however, the data in the variogram represents the best available information since the dataset was derived from a complete enumeration rather than samples. A large nugget can confuse model-fitting algorithms, and in the case of beech and Persian maple, the software-generated models appeared to overfit the structure and model a lower nugget and larger percent spatial structure than appears reasonable from visual inspection of the variogram. The roughly 20-m lag distances used in this study reflect some combination of large distances between trees for some species and the level of local variation in diameter present across the study area. Any spatial correlations over smaller distances or in specific directions were not captured in this variogram analysis. Given the large number of beech trees and a mean distance between trees of 3.9 m, further examination of beech in particular may reveal more detail regarding this species’ spatial patterns over such very small distances.

Field measurements can be expensive and time-consuming, particularly over large areas and in difficult terrain. One use of geostatistical methods in forest research has been to extract additional spatial information from available field data and add this information to modelled interpolations of conditions on the ground. Many of the results from these efforts have been encouraging in their ability to improve the accuracy of modeled estimates. In the complex topography of the Hyrcanian forest, a compound model would be necessary to capture both the short-range spatial structure and the longer-range cyclical structure. Anisotropic models are designed to capture directional differences in spatial structure. More research would be needed to better understand the correlations between spatial distribution of tree diameters to capture sufficient information on the spatial structure present in complex terrain. In this study, the results of variogram analysis showed that this method provided valuable information for characterizing the spatial distribution of diameter for beech, alder, linden, and Persian maple.

The Clark and Evans aggregation index results indicated that most species exhibited clumped spatial distributions, indicating a strong positive spatial structure in tree location. This information is consistent with the influence of catena type on species occurrence, but alone is insufficient for drawing conclusions. Variogram analysis of tree diameters by species clearly exhibited a cyclical spatial structure, indicating that a periodic feature of the Hyrcanian forest landscape, such as catena shape, was visibly affecting the diameter of three of the five most dominant species in the study area. Moreover, the variogram revealed a shorter-range spatial correlation of tree diameter for alder and linden, information which could be used to generate a model for geostatistical interpolation (e.g. kriging or conditional simulation) when only a sample of tree data is available. Such models could prove invaluable for investigating competitive interactions between species and various developmental stages within a single species which are the subject of much recent silvicultural research into mixed species multi-structured forests^25^.

Variogram analysis of species diameter made it possible to discern important additional details in the spatial structure of diameter distributions that were not available from local neighborhood analysis like the Clark and Evans aggregation index, even if it had been applied to other diameter classes. Variogram analysis provided information about the spatial correlation present over distances that could be related back to measurable features in the landscape and species-specific characteristics. This will prove an important tool in disentangling the effects of topography from other disturbances such as changes in climate etc.

## Conclusion

The Hyrcanian forests of northern Iran are recognised by UNESCO as being a world heritage site whose unique and strategic refugial biodiversity is severely threatened by disturbance pressures (https://whc.unesco.org/en/list/1584/). Its relatively inaccessible, steep, and complex catena terrain has made it challenging to evaluate species-terrain relationships. This study utilized a new approach for investigating complex forest landscape structures and their relationship to tree species distribution in a relatively straightforward manner. Variogram and aggregation analysis of Hyrcanian forest species indicated that several tree species in this relatively untouched beech forest exhibit^26^ both local and long-range spatial structure. Two of the five most common species exhibited local spatial correlation of tree diameters, and all five species exhibited clustered stem distributions, likely caused by exploiting a common regeneration opportunity. This builds on work in previous studies which identified that hornbeam trees tended to be separated from other tree species, often creating pure stands/groups, while beech and alder revealed a positive relationship with each other; indicative of complimentary growth, and resulted in mixed stands/groups^15^. Some comprehension of the spatial patterns of individual trees with respect to the catena topography is critical information for furthering our understanding of the ecology of these important ecosystems, as well as for practical management interested in using silvicultural treatments to conserve the composition and diversity of the local/regional natural forest in the context of current changing climate conditions. In this study we found that while the aggregation index provided an initial assessment of tree patchiness, variogram analysis of tree diameters provided substantial additional insight into mixed species stand structure and development, identifying an apparent scarcity of regeneration on the ground and in the understory. This anomaly in the younger age structure is probably indicative of excessive browsing pressure from ungulate species.

In the future, to evaluate the spatial attributes of diameter (and its insights into stand age/developmental stages) in other areas of the Hyrcanian forest it may not be necessary to measure distances or establish coordinates for all trees to generate accurate variograms for analysis of spatial structure. The spatial structure of stand tree diameters can also be evaluated from sample data. Additional research into the sampling intensity required for accurate variogram analysis would be valuable and practical, to further disentangle topographical impacts from other disturbance effects (e.g. using anisotropic variograms to evaluate cross-catena vs. within-catena beech, alder, and linden diameter distributions). Increased comprehension of the spatial patterns of Hyrcanian trees as they relate to topographic characteristics will not only improve our understanding of this important ecosystem in the face of future changes but will also increase the utility of topography-sensitive models to better predict species distribution in these complex landscapes.

## Supplementary Information

Supplementary Figures.
