# Supplementary material for: Fine-scale topographic influence on the spatial distribution of tree species diameter in old-growth beech (Fagus orientalis Lipsky.) forests, northern Iran
Source: Sci Rep. 2022 May 10;12:7633. doi: 10.1038/s41598-022-10606-0 (PMC9090739; doi:10.1038/s41598-022-10606-0)
Supplement: Supplementary file 1 — Supplementary Figures. [file 41598_2022_10606_MOESM1_ESM.docx]

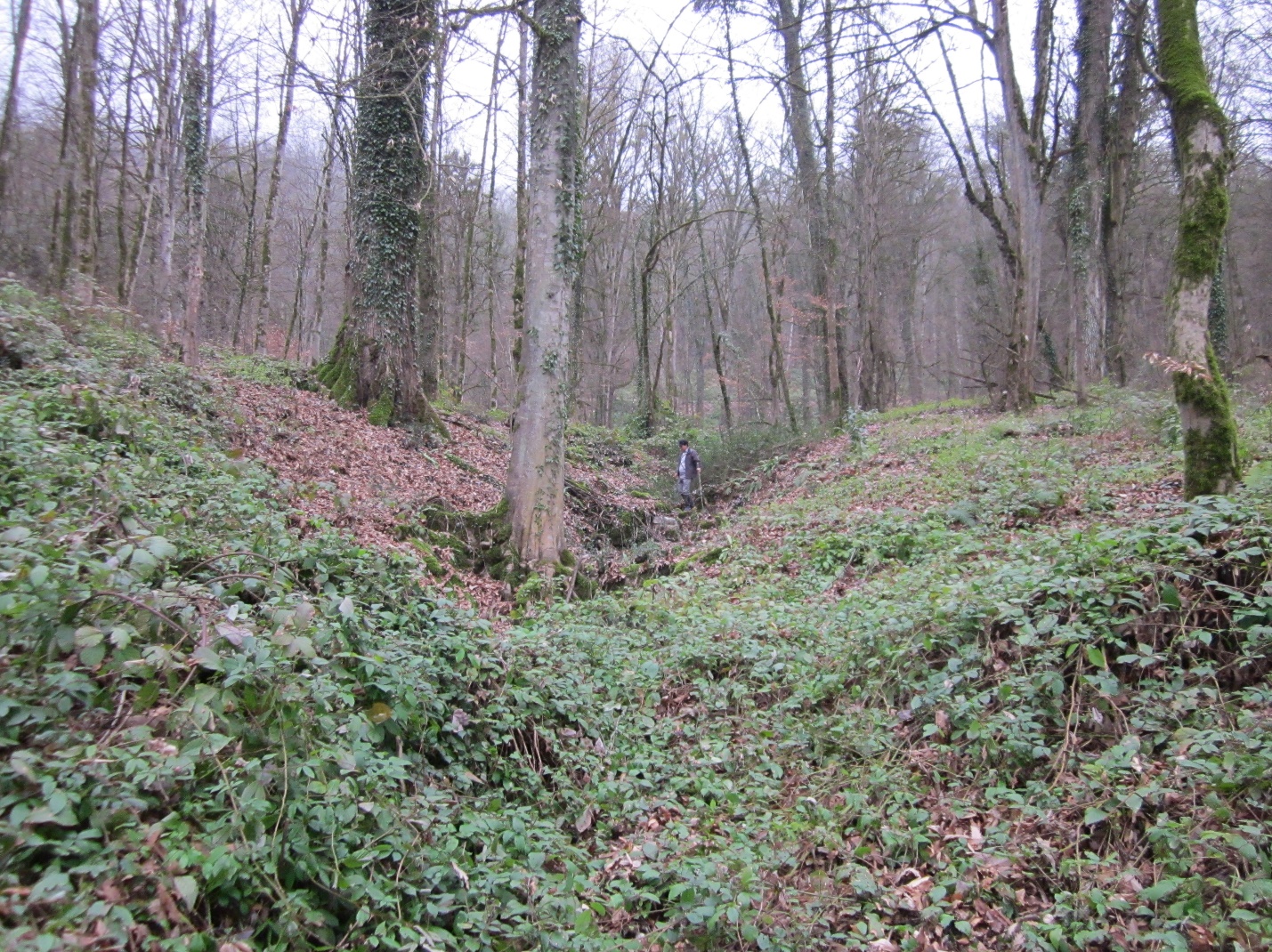


Figure S1. This is an example of a V-shaped catena at the Tarbiat Modares University research station. The relatively low stocking density is clearly apparent.


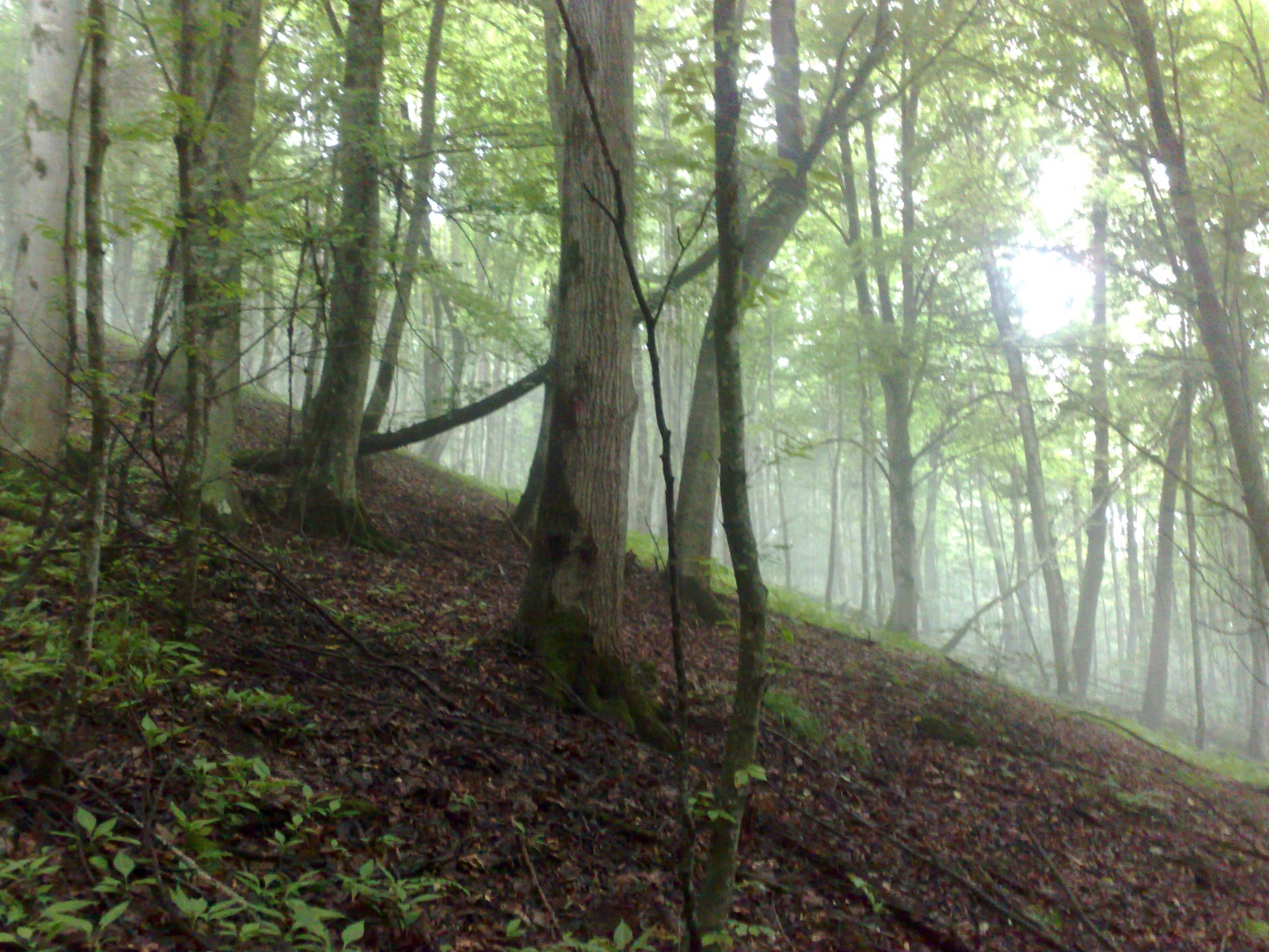


Figure S2. An example of a C-shaped catena. The lack of developed ground vegetation and tree species regeneration was typical of this area.
